# The Bioprospecting of *Bixa orellana* L. for the Selection of Characters with Biological Activity

**DOI:** 10.3390/metabo15020115

**Published:** 2025-02-10

**Authors:** Luz A. Guerrero-Lagunes, Lucero M. Ruiz-Posadas, Jorge Cadena-Iñiguez, Ramón Marcos Soto-Hernández, Carlos H. Avendaño-Arrazate, Juan F. Aguirre-Medina, Celeste Soto-Mendoza, Juan F. Aguirre-Cadena

**Affiliations:** 1 Colegio de Postgraduados, Campus Montecillo. Km. 36.5 Carretera Federal México-Texcoco, Montecillo, Texcoco 56264, Mexico; guerrero.luz@colpos.mx (L.A.G.-L.); msoto@colpos.mx (R.M.S.-H.); sotoalejandra1506@gmail.com (C.S.-M.); 2 Colegio de Postgraduados, Campus San Luis Potosí, Innovación en Manejo de Recursos Naturales, Iturbide 73, Salinas de Hidalgo, San Luis Potosí 78600, Mexico; jocadena@colpos.mx; 3 Instituto Nacional de Investigaciones Forestales, Agrícolas y Pecuarias, Centro Nacional de Recursos Genéticos, Blvd. de la Biodiversidad #400 Rancho las Cruces Tepatitlán de Morelos, Jalisco 47600, Mexico; avendano.carlos@inifap.gob.mx; 4 Faculty of Agricultural Sciences—Campus IV, Autonomous University of Chiapas, Junction of the Coastal Highway and the Town of Huehuetán, Chiapas 30660, Mexico; juan.aguirre@unach.mx (J.F.A.-M.); juan.cadena@unach.mx (J.F.A.-C.)

**Keywords:** achiote, biological activity, cancer

## Abstract

A meta-analysis of 28 sources of information was conducted, considering different variables in *Bixa orellana*, with the aim of identifying bioprospective variables. Variables were approached, such as the organ of extraction and extraction method, with 63 biochemical classes and 20 for biological activity, and their states were codified. The statistical analysis was developed through a cladistics analysis using the WinClada version1.00.08 84,85 software and the explicative accumulated variance was determined through a descriptive multivariate analysis and multiple correspondence analysis (MCA). The tree obtained showed the phenotype Africa1 as the one closest to the basal state. After Africa1, nine clades are derived and the phenotypes Colombia3 and Colombia5 were the most evolved. The analyses demonstrated that in *B. orellana* L., the phenotypes from India, Brazil, and Yucatán present anticancer activity against the cell lines U251, MCF-7, HeLa, NCI-H460, PC-3, A549 and HT-29, as well as biological activity against *Staphylococcus aureus*, *Escherichia coli*, and *Pseudomonas aeruginosa*, related primarily with biochemical compounds such as geranylgeraniol, ellagic acid, and carotenoids (bixin and norbixin), naringenin and alkaloids. The conditions of reproductive isolation of the phenotypes mentioned before providing the ideal agroclimatic conditions to produce compounds with biological activity.

## 1. Introduction

*Bixa orellana* L. (Bixaceae), known as achiote or annatto, is native to Central and South America and is grown in some tropical countries of the world, such as Peru, Mexico, Brazil, Colombia, Ecuador, Indonesia, India, Kenya, and Eastern Africa [1].

**Figure 1:**
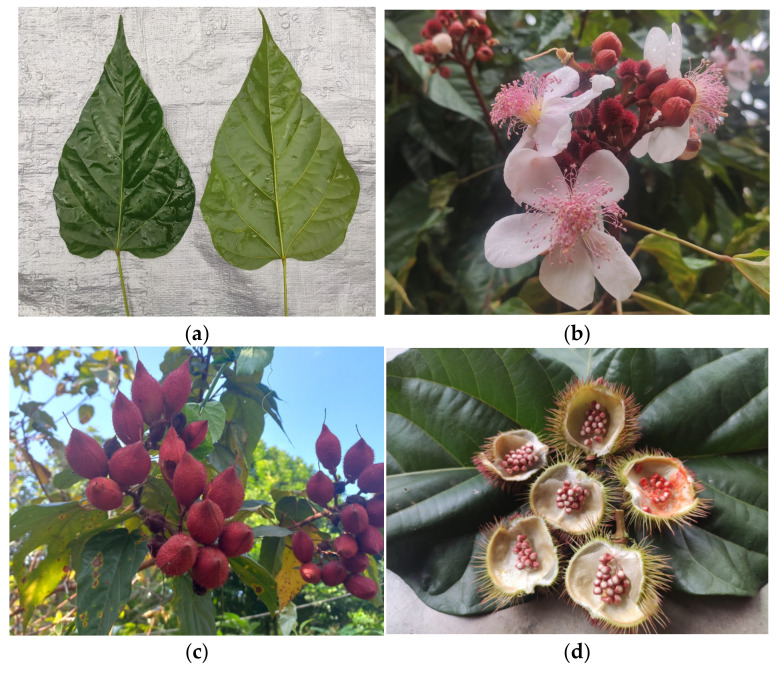
Leaf (**a**), flower (**b**), capsule (**c**), and seed (**d**) of *Bixa Orellana* L.

The plant is a shrub with simple ovate leaves and compound flowers, each consisting of five petals that may be white, violet, or pink. Its red, triangular seeds are enclosed in capsules covered with trichomes, as depicted in Figure 1. A morphological analysis of individuals from various origins has revealed significant phenotypic variability, manifested in traits such as leaf color and morphology, flower color, fruit shape, the presence of trichomes on the fruit, and seed count. These traits have been key in identifying and differentiating certain genotypes [2,3].

The seeds and leaves of *B. orellana* L. have been used since ancient times, both in culinary applications and traditional medicine. In the former, they are used to impart color and flavor, while in the latter, they are employed to treat a variety of ailments, including constipation, fever, heartburn, asthma, scabies, ulcers, and diarrhea [4]. The dyeing properties of the seeds, primarily attributed to the compounds bixin and norbixin, are currently exploited by several industries, including the food, textile, chemical, pharmaceutical, and cosmetics industries [5].

A range of bioactive compounds, including tannins, flavonoids, phenolic compounds, terpenoids, alkaloids, saponins, and anthraquinones, have been identified in the seeds and leaves [6,7,8]. The presence of bixin, norbixin, and geranylgeraniol in the seeds imparts biological activity with potential therapeutic applications [9,10,11].

The carotenoids, apocarotenoids, terpenes, terpenoids, sterols, and aliphatic compounds are the main compounds that are found in every part of this plant, for which a wide range of pharmacological activities have been researched [12]. Their biological activity has been demonstrated for the control of bacteria and fungi [10,13]. The antioxidant activity has been demonstrated by various studies [8,10,14], also displaying anticancer activity in cell lines of medical interest [9,11,15,16]; therefore, it has been included among nutraceutical foods. Because of its broad biological activity, *B. orellana* L. is a source for the development of new drugs with pharmacological activity, so there is the possibility of identifying morphological and phytochemical variables with a bioprospective approach, under the premise that the bioprospective meta-analysis facilitates the identification of the phenotype, its character, or outstanding phytochemical variable, as well as the state of the character, specifying the statistical validity and reducing possible contradictions in the literature.

## 2. Materials and Methods

An analysis of the studies published in the Scopus, Science Direct, Scifinder, Springer, and Google Scholar databases was carried out, using the search terms achiote, *B. orellana* L., phytochemicals, pharmacology, cancer, biological activity, antibacterial activity, and anticancer, as well as cytotoxic and antioxidant activity. From this, *n* = 56 results were identified, and when the criteria of plant organ and biological activity identified in each publication were applied, the sample was reduced to *n* = 28. All the studies included were studies that addressed the phytochemical characterization and biological activity of extracts from *B. orellana* L. (Table 1).

The studies included were conducted in Africa (*n* = 1), United States (*n* = 1), the Philippines (*n* = 1), Ecuador (*n* = 1), Bangladesh (*n* = 1), South Korea (*n* = 1), Nigeria (*n* = 2), Yucatán—Mexico (*n* = 2), Colombia (*n* = 5), India (*n* = 5), and Brazil (*n* = 6). The last three led the phytochemical and biological activity research of *B. orellana* L. The information was recorded in a database, codifying the variables and their different states (Table 2), made up by the following: organ of the plant used, extraction methods, biochemical classes, groups of compounds, phenols and phenolic acids, flavonoids, tannins, monoterpenes, sesquiterpenes, diterpenes, triterpenes, tetraterpenes, alkaloids, cyanogenic glucosides, and antimicrobial and anticancer activity.

### Statistical Analysis

Cladistic and statistical analyses were conducted based on the presence or absence of variables and their respective states (Table 2). Regarding biological and anticancer activity, the minimum inhibitory concentrations (MIC) of *B. orellana* L. extracts against *Pseudomonas aeruginosa*, *Esherichia coli*, *Staphylococcus aureus*, *Salmonella* sp., and *Candida albicans* are presented. The MIC obtained were 50 to 500, 50 to 1024, 50 to 1000, 1000 and 50 to 140 μg/mL, respectively. In terms of inhibition zones generated by the same extracts against these species, the reported values ranged from 13.00 to 100.00, 11.00 to 90.00, 11.00 to 100.00, 18.00 and 13.00 to 100.00 mm [1,10,12,15,16,18,19,20,22,27,29].

Furthermore, the anticancer activity of *B. orellana* L. extracts, assessed by their ability to reduce cell viability to 50% in various cancer cell lines, was observed at the following minimum concentrations: 100 µg/mL for liver cancer (HepG2), 3.9 µg/mL for glioblastoma multiforme (U251), 3.1 µg/mL for breast cancer (MCF-7), 37.2 µg/mL for cervical cancer (HeLa), 3.3 µg/mL for lung cancer (NCI-H460 and A549), 2.8 µg/mL for prostate cancer (PC-3), and 3.3 µg/mL for colon cancer (HT-29) [4,11,25,27,28].

Several authors [1,17,20] have demonstrated the antioxidant activity of *B. orellana*, reporting free-radical-scavenging DPPH IC_50_ values of approximately 1 to 15 mg/mL for seeds and 3.2 to 10 mg/mL for leaves.

The statistical analysis was developed with two approaches. The first through a cladistics analysis that incorporates the approach of Popper’s critical rationalism through the refutation of phylogenetic hypotheses examined under a parsimonious principle [31,32]; and through non-parametric statistics using the WinClada version 1.00.08 84,85 software (free license) [33], with the Bootstrap/Jackknife resampling methods, approaching the genotypes as populations through a random simulation until generating a parsimonious cladogram [34]. This analysis defines the stability of the clades and identifies the state of the outstanding variables. The analysis was repeated 1000 times, creating values such as support indices, consistency, and reliability in the cladograms [35]. The systematic reviews carried out in the meta-analysis were directed towards the information disseminated, to reanalyze it with approaches adapted to the present research [36]. It must be clarified that the criteria selected were those with complete, traceable data and reproducible results, to avoid biases in the study [37].

The second approach was to determine the explicative accumulated variance, the statistical weight of each variable, and its state through a descriptive multivariate analysis and multiple correspondence analysis (MCA), with the FactoMineR and factoextra [38] libraries with the Rstudio statistical package [39].

Following the phylogenetic and biochemical characterization of *B. orellana* L. phenotypes, a multivariate analysis using Principal Component Analysis (PCA) was performed to identify the key variables driving the differentiation of the chemical profile based on the extraction organ and the method applied. This analysis helped reduce the data complexity and group the biochemical, morphological, and functional traits into representative dimensions. The PCA applied to the 28 genotypes of *B. orellana* L. enabled the identification of the most significant variables responsible for the differentiation of the biochemical and functional profiles.

## 3. Results and Discussion

**Figure 2:**
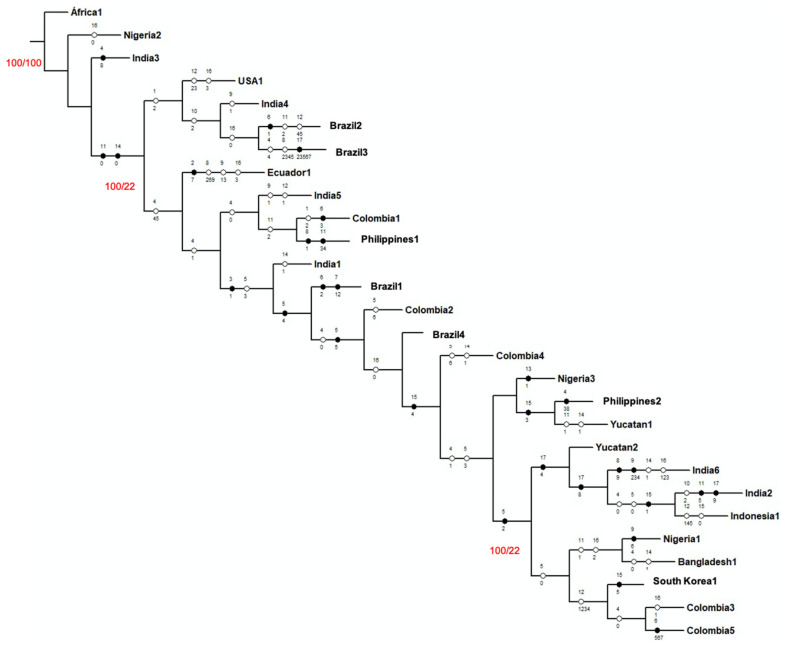
A cladogram of the phenotypes of *Bixa orellana* L. with different geographic origins, based on the plant organ used, extraction method, biochemical characters, and biological activity. White spots represent apomorphic variations and black spots plesiomorphic variations. The values separated by the diagonal line represent the Bootstrap/Jackknife indices, with L = 149, Ci = 50, and Ri = 64.

Figure 2 presents the general cladogram that indicates the distribution of the *B. orellana* L. phenotypes analyzed in function of the characters organ of extraction, method, biochemical class, and biological activity (Table 2). In total, 12 trees were obtained to create a consensus tree. This tree showed 149 steps or changes, a cladogram consistency index of 50%, and a retention index that reflects the percentage of characters that retain and conserve a change in taxa of 64%. The bioprospective meta-analysis presented in this paper aids in the identification of key phenotypic traits, variables, and their respective statuses, offering statistical validation while minimizing potential contradictions in the existing literature.

The parsimonious distribution of the phenotypes of *B. orellana* L. (Figure 2) is not indicative of a strict genealogical relationship, since there are no morphological and genetic characters; however, it helps to understand the adaptive specialization [40,41] of plants in the face of the differences in environmental conditions unlike those in their habitat. In general, reproductive isolation, selective pressure, and the lack of variability create unique survival characters reflected in the content and diversity of secondary metabolites [42].

The biochemical and biological activity variables showed that the phenotype with origin from Africa1 was located as the closest one to the basal state, hypothetically indicating due to the variables analyzed that it could have greater similarity with a phenotype from the original habitat (Figure 1).

Nine clades derive from Africa1. The first formed by Nigeria2 and India3, phenotypes closer to the root, which share the presence of alkaloids in leaves as plesiomorphic characters. Even when the publications do not record the time of introduction to Nigeria and India, it is presumed that they could have had some reproductive isolation, absence of variability, and agroclimatic conditions different from their geographic origin (Central and South America). Various authors mention how reproductive isolation and the absence of biological variation in some organisms promote unique characters that can be used in different sectors of society, such as in the case of enzymes responsible for producing secondary metabolites with biological effects of medical, agricultural, or industrial interest [43].

The second clade derives from India3, formed by the phenotypes USA1, India4, Brazil2, and Brazil3, which make up an independent evolutionary route characterized by sharing the seed as an organ of extraction, which is a derived state. The USA1 phenotype shares the presence of geranylgeraniol (apomorphic state) with the rest of the phenotypes from this group, highlighting that it has the derived characters cis-norbixin and trans-norbixin, which are related with the biological activity against *Staphylococcus aureus* (MIC 50 a 100 μg/mL), also a derived state. Brazil2 and Brazil3 are sibling phenotypes, and presumed to be those of greatest “evolved” specialization from this group, characterized by the presence of flavonoids, which is classified as an ancestral state. When it comes to apomorphic states, the presence of terpenes, ocimene, spathulenol, isoledene, and bergamotene stands out, as well as bixin and norbixin. This clade is categorized based on the organ of extraction and the presence of carotenoids within it. The phenotype Brazil3 shows anticancer activity, reducing cellular lines by 50% against the cell lines U251, MCF-7, NCI-H460, PC-3, and HT-29 from 3.9, 3.1, 3.3, and 3.3 μg/mL of *B. orellana* L. extract, and presents as a plesiomorphic state (ancient or primitive character). The distinction of the anticancer activity in a phenotype that is in the origin center of the species proves that the specific conditions of this place favored the presence of the compounds mentioned and contributed to the anticancer activity. However, when agroclimatic conditions change outside its place of origin, it loses chemical variability.

In the case of Ecuador1, it forms an independent clade and shows an evolutionary divergence, possibly due to reproductive isolation. A plesiomorphic state stands out in the group, which is an extraction method comprising the vapor sweeping of leaves, different from the rest of the genotypes, while the presence of ocimene, pinene, germacrene, farnesol and caryophyllene, as well as the activity against *Staphylococcus aureus*, are new characters or derived states (apomorphic). In this group, the extraction method marked the difference in the compounds detected.

The six remaining clades derive from Ecuador1. India5, Colombia1, and Philippines1 form a group characterized by the new or derived characters represented by the farnesol compounds, saponins, and carotenoids. The phenotypes from Colombia and the Philippines present three ancestral characters constituted by anthocyanins and polyprenol, as well as stigmaesterol and sitoesterol. Although both phenotypes do not have a geographic grouping, it is evident in the group of compounds, which indicates a possible displacement of the phenotypes from the center of origin towards the Philippines and India, where the original compounds could be conserved, or there was an influence of similar agroecological conditions that impacted the production of these secondary metabolites.

Also, Ecuador1 is derived from the group constituted by India1, Brazil1, Colombia2, Brazil4, and Colombia4. It should be mentioned that the phenotypes from Brazil are again those that present the highest number of plesiomorphic states (kaempherol, granatin, neostrictinin, procyanidines, and ellagic acid, as well as antioxidant activity). When it comes to apomorphic characters, the presence of saponins, tannic acid, and anthraquinones stands out, which are present in the phenotypes from Colombia and India. In this group, the geographic grouping is clear regarding the states that highlight the ancestral characteristics of the phenotypes from Brazil, the zone registered as the origin center of *B. orellana*.

Brazil4 is associated with anti-inflammatory activity and can be linked to the presence of ellagic acid, a finding that is in agreement with the authors of another study [44], who determined that this compound acts as chemo-protector against different types of cancer and shows strong antiproliferative activity against colon, lung, and prostate cancer cells.

From Colombia4, four subgroups are derived, an independent one formed by Nigeria3, Philippines2, and Yucatán1, which are characterized by presenting three plesiomorphic states represented by tannins, alkaloids, and atropines. From this group, the phenotype located in Yucatán is the most evolved, which is proven by the derived states present, such as the presence of saponins and the hepatoprotective activity. This evolution could be due to the agroclimatic characteristics or the manipulation of the crops in the zone, since in Yucatán there are commercial crops of *B. orellana* L. that have been genetically improved to reach higher seed production, which can be a factor that impacts the production of secondary metabolites [45].

The other sibling arm of Colombia4 groups, on the one hand, is Yucatán2, India6, India2, and Indonesia1, which, despite not having a geographic grouping, was characterized by the presence of the highest number of plesiomorphic states, among which germacrene, elemene, caryophyllene, and squalene stand out, as well as chemo-preventive activity. The anticancer activity of *B. orellana* L. extract, which reduces cell viability by 50%, was observed in the HeLa, A549, and MCF-7 cell lines at concentrations of 37.2, 2.8, and 3.1 µg/mL, respectively. This a simplesiomorphic state, since it also presents in Brazil3, which is a genotype close to the root.

The derived character has to do with the presence of geranylgeraniol, carotenoids, bixin, and norbixin, in addition to the activity against *P. aeruginosa*, *E. coli*, and *S. aureus*. The biological and anticancer activity is determined by the variety of phytochemical compounds present in *B. orellana* L. and by the capacity of geranylgeraniol to induce apoptosis in A549 cells [29,46,47]. In this group, it can be inferred that there was a flow of plants from Yucatán towards India and Indonesia and the antiproliferative activity was conserved.

The last two groups derive from the branch coming from Colombia4. The node formed by Nigeria1 and Bangladesh share the apomorphic states represented by saponins and the biological activity against *E. coli* (MIC of 50 to 1024 μg/mL). Only an ancestral state is present (tomentosin).

The phenotypes SouthKorea1, Colombia3, and Colombia5 are the last group and share four apomorphic states integrated by carotenoids, cis-norbixin, trans-norbixin, and bixin. In addition, they present butein, catechins, and chlorogenic acid, as well as cytotoxic activity, as ancestral characters. The phenotypes located in Colombia were considered the most evolved, compared to Africa1, Nigeria2, and India3, whose evolution can be due to pressure processes, such as manipulation, edaphoclimatic conditions, or the genetic flow between genotypes. It should be highlighted that the activity found in the phenotypes present in this bioprospective study is consistent with that found by other authors [1,48,49], who determined that the tannins, quinones, and terpenoids have biological activity; in addition, lipophylic flavonoids can be disruptive for the cell membrane [49].

Table 3 shows the apomorphic characters present in the phenotypes studied, observing sinapomorphic characters (shared characters) among the phenotypes USA1, India2, and India4, such as geranylgeraniol, while India4 and India5 share farnesol; Brazil2 and India5, steroids; Colombia2 and Colombia4, tannic acid; Colombia4, India6, and Bangladesh1, saponins; India5 and Indonesia1, carotenoids; Brazil2 and Indonesia1, bixin and norbixin; Brazil3 and Ecuador1, ocimene; USA1 and Ecuador1, activity against *S. aureus*; and India6 and Colombia3 activity against *P. aeruginosa*. On the other hand, cis-norbixin, trans-norbixin, spathulenol, isoledene, bergamotene, germacrene, farnesol, and caryophyllene are autopomorphic states (unique characters) because they are present in a single taxon or genotype.

Table 4 also shows that apomorphic characters (new or derived) are related to the antimicrobial activity against *P. aeruginosa, E. coli*, *S. aureus*, and biochemical class, primarily the carotenoids bixin, norbixin, 9′-cis-norbixin, transnorbixin, saponins, and monoterpenes. The plesiomorphic characters are more closely related to the hepatoprotective, chemo-preventive and anticancer activity against the cell lines MCF-7, NCI-H460, PC-3, HT-29, HeLa and A549, as well as the presence of flavonoids (naringenin, kaempherol, anthocyanins, procyanidines, ellagic acid), triterpenes (stigamesterol and sitoesterol), tannins (granatin, neostrictinin), sesquiterpenes (elemene, caryophyllene), and coumarins. It stands out that some compounds from *B. orellana* in this bioprospective analysis act on microorganisms that can cause public health problems, highlighting characters for a possible program for genetic improvement.

It is important to highlight that the non-detection of a compound does not mean it is absent, since it could have remained undetected because of the plant organ used, extraction method, or the seasonal time of sample harvesting. For future studies, our proposal is to elucidate the “absent” compounds to secure the grouping; something to remember is that since this is a meta-analysis, comparative biases between taxa can occur due to the various methods of sampling, extraction, and analysis. Over-studied and under-studied taxa can bring biases to the analysis.

### Multivariate Analysis

The multivariate analysis allowed us to identify the variables that explain the highest explicative variance, in addition to exploring the correlations and reducing the dimension of the analysis with new indices [50]. It was determined that in four principal components (PCs), the accumulated value is 86.31% (Table 5).

As shown in Table 6, the extraction organ had a distinct impact on the biochemical variability of the phenotypes. Leaves exhibited a higher factor loading in PC1 (0.40600), indicating that this tissue concentrates a significant proportion of metabolites relevant to the differentiation of chemical profiles. In contrast, seeds contributed to a lesser extent, with a lower loading in PC2 (0.09092), suggesting that while their chemical composition is unique, their impact on the overall variability is less influential.

The extraction method revealed differences in the recovery of bioactive compounds. Ethanol extraction showed a significant loading in PC1 (0.07360), suggesting higher efficiency in recovering key metabolites, particularly carotenoids and flavonoids.

Biochemical classes exhibited clear differentiation among phenotypes. Terpenoids were particularly prominent, with loadings in both PC1 (0.18442) and PC2 (0.20710), highlighting their structural role in the chemical variability of *B. orellana* L. Principal component analysis also revealed the influence of secondary metabolites of *B. orellana* L., showing a clear differentiation between phenotypes in the profiles of bioactive compounds, as outlined in the table. Flavonoids stood out with a significant factor loading in CP1 (0.29988), reflecting their strong association with the observed chemical variability, indicating that they play a key role in phenotype differentiation. On the other hand, tannins, with a prominent contribution in CP3 (0.20160), serve a differentiating role in a secondary dimension of biochemical variability. Terpenoids, with notable loadings in CP1 (0.18442) and CP2 (0.20710), further confirm their significant impact on the chemical composition of phenotypes, underscoring their contribution to the observed chemical variability.

The biological activity of *B. orellana* L. was assessed in terms of its anticancer and antimicrobial potential, with a focus on its relationship to the chemical profiles of the phenotypes. The results show a strong correlation between antiproliferative activity and the MCF-7 (0.47323) and HeLa (0.45618) cell lines in CP1, indicating that these cell lines are key differentiators between phenotypes. This association suggests that certain phenotypes exhibit a stronger antiproliferative effect, highlighting their potential for future pharmacological research.

Regarding antimicrobial activity, the relationship with *E. coli* (0.21822) and *P. aeruginosa* (0.16510) in CP2 and CP1, respectively, indicates a moderate association between antimicrobial activity and the biochemical differentiation of phenotypes. These findings suggest that *B. orellana* L. phenotypes could have potential for antimicrobial applications, particularly against these bacteria. In contrast, the activity against *C. albicans* showed less influence on the overall data structure, suggesting its impact on the observed variability is less significant.

Overall, it is observed that anticancer activity is influenced by the presence of bixin and norbixin; however, the results also suggest that these compounds are not the sole determinants. Synergistic interactions with flavonoids and alkaloids may also be contributing to the observed activity, emphasizing the complexity of the mechanisms involved.

## 4. Conclusions

There is scientific evidence for the use of *B. orellana* L. as an agent with anticancer activity, primarily against the cell lines U251, MCF-7, HeLa, NCI-H460, PC-3, A549, and HT-29, as well as biological activity against *S. aureus*, *E. coli*, and *P. aeruginosa*. The antimicrobial and anticancer activity is related primarily to biochemical compounds such as geranylgeraniol, ellagic acid, carotenoids (bixin and norbixin), naringenin, and alkaloids. The conditions of reproductive isolation of the phenotypes from Brazil, Yucatán, India, and Indonesia provided the ideal agroclimatic conditions to produce compounds with biological activity, because they produce those metabolites. This analysis can be used as reference for additional studies, genetic improvement programs, and the revaluation of the species.

## Figures and Tables

**Table 1:** Publications included in the bioprospecting meta-analysis in *Bixa orellana* L.

Variable	Phenotype	Research Focus	References
Biological activity	África1; USA1; Colombia1; Philippines1; India1; Colombia 2; Colombia3; India2; Nigeria1; South Korea1; India3; Colombia4; Philippines2; Yucatán1; Brazil1; Bangladesh1; Brazil2; Colombia5; Brazil3; India4; Indonesia1; Brazil5; India5; Yucatán2; Ecuador1	Antimicrobial, anticancer, antioxidant and hepatoprotective activity of leaves and/or seeds of *Bixa orellana*	[1,8,9,10,11,13,14,15,16,17,18,19,20,21,22,23,24,25,26,27,28,29,30]
Biochemistry	África1; USA1; Colombia1; Philippines1; India1; Colombia2; Colombia3; India2; Nigeria1; South Korea1; Nigeria2; India3; Colombia4; Philippines2; Yucatán1; India4; Colombia5; Brazil3; India5; Yucatán2; Ecuador1	Phytochemical characterization of extracts and essential oil of leaves and seeds of *Bixa orellana*	[1,7,8,9,10,11,12,13,14,15,16,17,18,19,20,21,22,23,24,25,26,27,28,29,30]

**Table 2:** Characters and character states of *Bixa orellana* L., for the bioprospecting analysis.

Number	Variable	Variable Status *
1	Extraction organ	Leaves = 1, Seeds = 2
2	Extraction method	Absent = 0, Aqueous and ethanolic extracts = 1, Solvent system = 2, Methanol = 3, Ethanol = 4, Petroleum ether = 5, Maceration = 6, Steam Distillation = 7
3	Biochemistry class	Absent = 0, Phenolic compounds = 1, Terpenoids = 2, Compounds with nitrogen = 3
4	Compound group	Phenols and phenolic acids = 1, Flavonoids = 2, Tannins = 3, Monoterpenoids and sesquiterpenoids = 4, Diterpenes = 5, Triterpenoids = 6, Tetraterpenoids = 7, Alkaloids = 8, Cyanogenic glycosides = 9
5	Phenols and phenolic acids	Absent = 0, Phenylpropanoids = 1, Coumarins = 2, Anthraquinones = 3, Procyanidins = 4, Ellagic acid = 5, Tannic acid = 6, Gallic acid = 7
6	Flavonoids	Absent = 0, Naringenin = 1, Kaemferol = 2, Anthocyanins = 3, Isoflavonoids = 4, Butein = 5, Catechins = 6, Chlorogenic acid = 7, Hypolaetin = 8
7	Tannins	Absent = 0, Granatin = 1, Neostrictinin = 2, Ellagitanin = 3
8	Monoterpenes	Absent = 0, Poliprenol = 1, Ocimene = 2, Spathulenol = 3, Isoledene = 4, Bergamotene = 5, Pinene = 6, Aristolochene = 7, Cadinene = 8, Germacrene = 9
9	Sesquiterpenes	Absent = 0, Farnesol = 1, Elemene = 2, Caryophyllene = 3, Guaiol = 4, Tomentosin = 5, Ishwarane = 6
10	Diterpenes	Absent = 0, Phytol = 1, Geranylgeraniol = 2, Geranyl terpinene = 3, Geranyl linalool = 4, Farnesyl = 5
11	Triterpenes	Absent = 0, Saponins = 1, Steroids = 2, Stigmasterol = 3, Sitosterol = 4, Squalene = 5
12	Tetraterpenes	Absent = 0, Carotenoids = 1, 9′-cis-norbixin = 2, Trans-norbixin = 3, Bixin = 4, Norbixin = 5, Diapocarotenoids = 6
13	Alkaloids	Absent = 0, Atrophin = 1
14	Cyanogenic glycosides	Absent = 0, Saponins = 1
15	Biological activity	Absent = 0, Chemo preventive = 1, Anti-inflammatory = 2, Hepatoprotective = 3, Antioxidants = 4, Cytotoxic = 5
16	Antimicrobial activity	Absent = 0, *Pseudomonas aeruginosa* = 1, *Escherichia coli* = 2, *Staphylococcus aureus* = 3, *Salmonella* sp. = 4, *Candida albicans* = 6
17	Anticancer activity	Absent = 0, HepG2 = 1, U251 = 2, MCF-7 = 3, HeLa = 4, NCI-H460 = 5, PC-3 = 6, HT-29 = 7, A549 = 8, MCF-7 = 9

* 0–9 variable statuses assigned in the cladistic (WinClada) and multivariate analysis.

**Table 3:** Apomorphic characters (new or derived) from the heuristic clade with biochemical and biological activity variables in 28 genotypes of *Bixa orellana* L. The diagonal line indicates the character and its relevant character status.

Phenotype	Character/Character State	Biochemistry	Biological Activity/Antimicrobial Activity/Anticancer Activity
Nigeria2			
India3			
USA1	10/2, 12/2, 12/3, 16/3,	Geranylgeraniol, cis-norbixin, trans-norbixin	*Staphylococcus aureus*
India4	9/1, 10/2,	Farnesol, geranylgeraniol	
Brazil2	11/2, 12/4, 12/5	Steroids, bixin, norbixin	
Brazil3	4/4, 8/2, 8/3, 8/4, 8/5	Mono- and sesquiterpenoids, ocimene, spathulenol, isoledene, bergamotene	
Ecuador1	8/2, 8/6, 8/9, 9/1, 9/3, 16/3	Ocimene, pinene, germacrene, farnesol, caryophyllene	*Staphylococus aureus*
India5	9/1, 11/2, 12/1	Farnesol, saponins, carotenoids	
Colombia1	11/2	Saponins	
Philippines1			
India1	14/1	Saponins	
Brazil1			
Colombia2	5/6	Tannic acid	
Brazil4			
Colombia4	5/6, 14/1	Tannic acid, saponins	
Nigeria3			
Philippines2			
Yucatán1	11/1	Saponins	
Yucatán2			
India6	14/1, 16/1, 16/2, 16/3	Saponins	*Pseudomonas aeruginosa*, *Escherichia coli*, *Staphylococus aureus*
India2	10/2	Geranylgeraniol	
Indonesia1	12/1, 12/4, 12/5	Carotenoids, bixin, norbixin	
Nigeria1			
Bangladesh1	14/1	Saponins	
South Korea1			
Colombia3	16/1		*Pseudomonas aeruginosa*
Colombia5			

**Table 4:** Apomorphic and plesiomorphic characters identified in the branches of the heuristic clade based on biochemical variables of the biological activity of *Bixa orellana* L. The diagonal line indicates the character and its relevant character status.

Branch	Apomorphic Character	Branch	Plesiomorphic Character
1		1	Alkaloids
2	Seeds, mono- and sesquiterpenoids, ocimene, spathulenol, isoledene, bergamotene, farnesol, saponins, geranylgeraniol, cis-norbixin, trans-norbixin, bixin, norbixin, *Staphylococcus aureus*	2	Naringenin, U251, MCF-7, NCI-H460 PC-3 and HT-29 cell lines
3	Phenols and phenolic acids, mono- and sesquiterpenoids, diterpenes, ocimene, pinene, germacrene, farnesol, caryophyllene, *Staphylococcus aureus*	3	Steam distillation
4	Seeds, phenols and phenolic acids, farnesol, steroids, carotenoids	4	Anthocyanins, phenylpropanoids, stigmasterol, sitosterol
5	Phenols and phenolic acids, anthraquinones, tannic acid, saponins	5	Phenolic compounds, procyanidins, ellagic acid, kaempherol, granatin, neostrictinin, antioxidant
6	Saponins	6	Tannins, alkaloids, atrophin, hepatoprotective
7	Geranylgeraniol, carotenoids, bixin, norbixin, *Pseudomonas aeruginosa*, *Escherichia coli*, *Staphylococcus aureus*	7	Coumarins, germacrene, elemene, caryophyllene, squalene, chemo preventive, HeLa cell lines, A549, MCF-7
8	Saponins, *Staphylococcus aureus*	8	Coumarins, caryophyllene
9	Carotenoides, cis-norbixina, trans-norbixina, bixina, *Pseudomonas aeruginosa*	9	Coumarins, butein, catechins, chlorogenic acid, cytotoxic

**Table 5:** Characteristic values and proportion accumulated for four principal components of the analysis of 28 phenotypes of *Bixa orellana* L. with different geographic origins, based on the organ of the plant used, extraction method, biochemical characters, and biological activity.

PC	Eigenvalues	Variance	Cumulative Variance	%
1	0.1046	0.0963	0.0963	9.63
2	0.0963	0.0887	0.1851	28.14
3	0.0717	0.0727	0.2578	53.92
4	0.0717	0.0660	0.3239	86.31

**Table 6:** Characteristic vectors of the analysis of 28 phenotypes of *Bixa orellana* L. with different geographic origins, based on the organ of the plant used, the extraction method, biochemical characters, and biological activity.

Variable	Variable States	CP1	CP2	CP3	CP4
Extraction organ	Leaves	**0.40600**	0.02947	0.00653	0.00442
	Seeds	**0.16010**	**0.09092**	0.00378	0.02480
Extraction method	Aqueous and ethanolic extracts	**0.08108**	0.00111	0.04358	0.00607
	Solvent system	0.00741	0.00181	**0.14477**	0.01652
	Methanol	0.00011	0.01826	0.00144	0.00843
	Ethanol	**0.07360**	0.03881	0.00227	0.04915
	Petroleum ether	0.01204	0.00217	0.00390	0.01648
	Maceration	0.00519	**0.29127**	0.06014	0.00480
	Steam distillation	0.00519	**0.52403**	**0.09163**	0.00072
Biochemistry class	Phenolic compounds	0.00470	0.01508	0.00001	0.00259
	Terpenoids	**0.18442**	**0.20710**	0.05018	**0.17695**
	Compounds with nitrogen	0.01603	0.00538	0.00219	**0.16758**
Compound group	Phenols and phenolic acids	**0.11318**	0.00859	0.00795	0.01836
	Flavonoids	**0.29988**	**0.12431**	**0.16761**	**0.13349**
	Tannins	**0.26420**	**0.12683**	**0.20160**	**0.15101**
	Monoterpenoids and sesquiterpenoids	0.00000	0.00000	0.00000	0.00000
	Diterpenes	0.00000	0.00000	0.00000	0.00000
	Triterpenoids	**0.18557**	0.06410	**0.08623**	**0.09697**
	Tetraterpenoids	0.04881	0.00190	0.00214	0.01425
	Alkaloids	**0.26420**	**0.12683**	**0.20160**	**0.15101**
	Cyanogenic glycosides	0.00000	0.00000	0.00000	0.00000
Phenols and phenolic acids	Phenylpropanoids	**0.15877**	0.06654	**0.12130**	**0.13140**
	Coumarins	**0.21741**	0.02699	0.00046	**0.35100**
	Anthraquinones	**0.26599**	**0.07780**	0.04298	**0.14762**
	Procyanidins	0.00656	0.00017	0.00004	0.06603
	Ellagic acid	0.00002	0.00002	0.00005	0.02223
	Tannic acid	0.00108	0.00116	0.00310	0.04040
	Gallic acid	0.00000	0.00000	0.00000	0.00000
Flavonoids	Naringenin	**0.40034**	0.02715	0.02587	**0.09716**
	Kaemferol	0.00272	0.00312	0.00331	**0.10585**
	Anthocyanins	0.00059	0.00027	0.01002	0.00397
	Isoflavonoids	0.00000	0.00000	0.00000	0.00000
	Butein	0.00013	0.00063	0.00117	**0.08565**
	Catechins	0.00000	0.00943	0.00020	0.03638
	Chlorogenic acid	0.01195	0.00235	0.03611	0.05006
	Hypolaetin	0.00000	0.00000	0.00000	0.00000
Tannins	Granatin	0.04169	0.03330	0.06398	0.01294
	Neostrictinin	0.00000	0.00000	0.00000	0.00000
	Ellagitanin	0.00000	0.00000	0.00000	0.00000
Monoterpenes	Poliprenol	0.04622	0.01371	**0.20700**	**0.28666**
	Ocimene	0.00098	**0.77755**	**0.17288**	0.00777
	Spathulenol	0.00098	**0.77755**	**0.17288**	0.00777
	Isoledene	0.00000	0.00000	0.00000	0.00000
	Bergamote	0.00000	0.00000	0.00000	0.00000
	Pinene	0.00098	**0.77755**	**0.17288**	0.00777
	Aristolochene	0.00000	0.00000	0.00000	0.00000
	Cadinene	0.00000	0.00000	0.00000	0.00000
	Germacrene	0.00098	**0.77755**	**0.17288**	0.00777
Sesquiterpenes	Farnesol	0.02186	**0.38639**	**0.25792**	**0.23356**
	Elemene	0.00098	**0.77755**	**0.17288**	0.00777
	Caryophyllene	0.00098	**0.77755**	**0.17288**	0.00777
	Guaiol	0.00000	0.00000	0.00000	0.00000
	Tomentosin	0.00000	0.00000	0.00000	0.00000
	Ishwarane	0.00031	0.02955	**0.35986**	0.01434
Diterpenes	Phytol	0.00247	0.01756	**0.09808**	0.08027
	Geranyl geraniol	0.00183	0.00469	0.34644	0.04852
	Geranyl terpinene	0.01807	0.02596	**0.47671**	**0.14103**
	Geranyl linalool	0.00011	0.01908	**0.33076**	0.00082
	Farnesyl	0.00011	0.01908	**0.33076**	0.00082
Triterpenes	Saponins	**0.10829**	0.00292	0.00052	0.02278
	Steroids	0.00186	0.01413	0.00282	0.00832
	Stigmasterol	**0.10291**	**0.07116**	**0.16746**	**0.11540**
	Sitosterol	**0.19469**	0.00139	0.00820	**0.14306**
	Squalene	**0.12040**	0.00217	0.00390	0.01648
Tetraterpenes	Carotenoids	**0.40393**	0.02718	0.03930	**0.09930**
	9′-cis-norbixin	0.01080	0.00040	0.00393	0.01509
	Trans-norbixin	0.01080	0.00040	0.00393	0.01509
	Bixin	**0.48865**	0.04809	0.03452	0.02605
	Norbixin	**0.48865**	0.04809	0.03452	0.02605
	Diapocarotenoids	0.00000	0.00000	0.00000	0.00000
Alkaloids	Atrophin	**0.21741**	0.02699	0.00046	**0.35100**
Cyanogenic glycosides	Saponins	0.00000	0.00000	0.00000	0.00000
Biological activity	Chemo-preventive	0.00568	0.00568	0.02083	0.06208
	Anti-inflammatory	0.00000	0.00000	0.00000	0.00000
	Hepatoprotective	0.00544	0.00644	0.00785	0.02204
	Antioxidants	0.02597	0.05981	**0.07794**	0.05050
	Cytotoxic	0.00000	0.00000	0.00000	0.00000
Antimicrobial activity	*Pseudomonas aeruginosa*	**0.16510**	0.00000	**0.14469**	**0.08732**
	*Escherichia coli*	**0.08361**	0.01917	**0.35559**	0.02388
	*Staphylococcus aureus*	0.05017	**0.21822**	0.00890	0.03508
	*Salmonella* sp.	**0.13572**	0.04070	0.02404	0.00446
	*Candida albicans*	**0.17932**	0.01001	0.03820	**0.13449**
Anticancer activity	HepG2	0.00000	0.00000	0.00000	0.00000
	U251	**0.45618**	0.02193	0.05048	0.22606
	MCF-7	**0.47323**	0.02313	0.03783	**0.16672**
	HeLa	**0.43024**	0.00843	0.00906	0.00554
	NCI-H460	0.04561	0.02193	0.05048	**0.22606**
	PC-3	**0.45618**	0.02193	0.05048	**0.22606**
	HT-29	**0.45618**	0.02193	0.05048	**0.22606**
	A549	0.00048	0.00056	0.03869	**0.15758**
	B16F10	0.00000	0.00000	0.00000	0.00000

The values highlighted in bold letters are statistically significant by principal component.

## Data Availability

Data are contained within the article.
